# Nurses’ Perceptions of the Scope of Nursing Support for Offense-Related Self-Reflection Beyond Formal Programs and Dedicated Interviews in Japanese Forensic Psychiatric Care

**DOI:** 10.3390/nursrep16090313

**Published:** 2026-09-02

**Authors:** Jun Okuda, Yoshiyuki Takashima

**Affiliations:** Department of Psychiatric and Mental Health Nursing, Faculty of Nursing, School of Medicine, Nara Medical University, Nara 634-0813, Japan

**Keywords:** forensic mental health care, Medical Treatment and Supervision Act, offense-related self-reflection, nursing support, recidivism prevention, psychiatric nursing, nurses’ perceptions, qualitative content analysis

## Abstract

**Background/Objectives:** Nurses may support offense-related self-reflection through practices that extend beyond formal group programs and dedicated offense-focused interviews; however, the scope of such support remains unclear. In this study, we described the range of nursing practices that nurses in Japanese forensic psychiatric inpatient settings perceived as supporting offense-related self-reflection beyond these formal interventions. **Methods:** A qualitative descriptive study was conducted using individual semi-structured interviews with 13 nurses from six hospitals between May and November 2025. Japanese-language transcripts were analyzed using inductive qualitative content analysis. Meaning units were coded and iteratively organized into subcategories and categories. Credibility and dependability were strengthened through co-researcher review, reflexive discussion, audit trail, and peer debriefing. **Results:** Five categories and 15 subcategories were identified: (1) examining offenses, victim impact, and responsibility; (2) clinical and relational readiness for self-reflection; (3) applying reflective insights to recidivism-prevention actions; (4) extending reflective learning into post-discharge life planning; and (5) integrating offense-related understanding into inpatient–community care planning. **Conclusions:** The categories describe functions that participants attributed to nursing practices surrounding formal interventions; therefore, they should not be interpreted as validated components of self-reflection or as evidence of effectiveness. The findings provide an initial, context-specific framework to guide nursing assessment, care planning, education, and further conceptual and empirical research that includes patients and community providers.

## 1. Introduction

Forensic mental health care combines treatment and rehabilitation with a responsibility to safeguard public safety. Care often occurs in long-term, restrictive settings; thus, patients’ interests, legal responsibilities, risk management, and ethical obligations intersect [1]. This dual mandate creates clinical and ethical demands not typically encountered in general psychiatric care.

Offense-related therapeutic work helps patients examine the offense and its contributing circumstances and link this insight to strategies for preventing reoffending. The risk–need–responsivity (RNR) model prioritizes risk level, criminogenic needs, and individual responsivity [2]. Forensic guidance and empirical research similarly emphasize addressing offense-related factors within treatment [3,4]. The good lives model (GLM) broadens this focus to meaningful and prosocial living [5,6], whereas recovery-oriented perspectives emphasize hope, self-determination, connectedness, meaning, identity, and empowerment alongside symptom and risk management [7,8].

Formal offense-specific group programs remain important, even as their evidence base and integration into routine practice are still evolving [9]. Research indicates that forensic mental health nursing encompasses several interconnected domains. Nurses have described addressing the offense therapeutically within the nurse–patient relationship [10], maintaining and rebuilding therapeutic relationships in secure settings [11,12], supporting patient engagement [13], contributing to shared risk assessment and management [14], and integrating the offense, risk, legal context, and therapeutic relationships into clinical reasoning [15]. Contemporary recovery research also highlights hope, identity, participation, and opportunities for daily life in forensic care [16,17,18]. However, these domains have largely been analyzed in isolation, leaving it unclear how nurses define the boundaries of practices that support offense-related self-reflection outside formal interventions.

In Japan, the Medical Treatment and Supervision Act (MTSA) established specialized inpatient and outpatient pathways for individuals with mental disorders who have committed serious offenses, with treatment and coordinated social reintegration as central aims [19]. Nurses in MTSA inpatient wards provide continuous care, participate in structured programs and individual work, observe how patients apply their learning in everyday ward life, and contribute to multidisciplinary discharge planning. Japanese community-based research has described nursing strategies related to risk assessment, offense-related work, transitional care, legal understanding, and multidisciplinary coordination [20]. A recent national overview has further detailed the coordinated outpatient pathway under the MTSA [21]. Because our literature review was narrative rather than systematic, we do not claim that no comparable study exists. However, we did not find any empirical studies specifically examining how nurses in Japanese MTSA inpatient wards define support for offense-related self-reflection outside formal group programs and dedicated offense-focused interviews.

Accordingly, we aimed to describe the range of nursing practices that nurses perceived as supporting offense-related self-reflection outside formal group programs and dedicated offense-focused interviews in Japanese forensic psychiatric inpatient settings. The central research question was: what nursing practices do nurses in Japanese MTSA inpatient wards perceive as supporting offense-related self-reflection outside these formal interventions?

## 2. Materials and Methods

### 2.1. Study Design and Working Definition

The working definition for this study was developed based on the research aim, the term’s use in Japanese clinical practice, and the existing literature on offense-related therapeutic work [3,4,10]. “Offense-related self-reflection” referred to the process through which a patient examines the offense, its antecedents and consequences, and its personal meaning. “Support for offense-related self-reflection” (hereafter, “support for self-reflection”) referred to nursing practices that participants perceived as facilitating this process outside formal group programs and dedicated offense-focused interviews. This provisional definition was not a validated construct. Offense awareness, insight, accountability, and responsibility were treated as possible components or outcomes of reflection rather than as synonyms for the process. Risk management, rehabilitation, recovery, discharge planning, and community reintegration were treated as broader care domains. Activities within these domains were included only when participants explicitly linked them to facilitating, deepening, applying, or sustaining offense-related self-reflection.

Within the participating MTSA inpatient wards, “formal offense-related programs” referred to scheduled, structured group sessions explicitly focused on the offense and related factors. “Dedicated offense-focused interviews” referred to planned one-to-one sessions with the same explicit purpose. Depending on the facility and treatment phase, nurses facilitated or co-facilitated sessions, reviewed program content with patients, observed how patients applied this learning in daily ward life, and integrated these observations into multidisciplinary treatment and discharge planning. In this study, we examined the nursing practices surrounding these formal interventions rather than their content.

A qualitative descriptive design was selected to provide a clinically accessible, low-inference account of how nurses understood this area of practice. Because the study was not intended to generate a formal theory or elucidate the essence of an experience, it adopted a naturalistic and pragmatic orientation: participants’ accounts were treated as contextually situated descriptions, and the analysis remained close to their language while allowing limited abstraction for category development [22]. Reporting adhered to the Standards for Reporting Qualitative Research [23].

### 2.2. Setting, Recruitment, and Participants

Facilities were selected using convenience sampling based on recruitment feasibility. The researchers contacted directors of nursing by telephone or email. After receiving an explanation of the study and confirming that their wards provided support for self-reflection, the directors identified nurses who meet the eligibility criteria and introduced them to the researchers rather than distributing a general invitation. Researchers then provided study information directly to each nurse, confirmed eligibility, and obtained consent independently of the directors. Six hospitals participated. Nurses were eligible if they were currently providing direct care in an MTSA inpatient ward and had experience supporting self-reflection; managers not involved in direct care were excluded. Purposive maximum-variation or stratified sampling was not used. Variation in hospital, age, gender, and years of psychiatric and forensic nursing experience arose through multisite convenience recruitment rather than predetermined quotas. Clinical roles and experience with specific program formats were not used as sampling strata. Thirteen nurses participated in total.

### 2.3. Data Collection

The semi-structured interview guide was developed to address the central research question, drawing on four key sensitizing themes: (1) the MTSA aims of social reintegration and prevention of further serious harm; (2) Japanese and international literature on offense-related work and forensic nursing [4,10,11,12,13,14,15,20]; (3) the continuous nature of psychiatric nursing, which encompasses care in everyday living situations; and (4) qualitative descriptive principles emphasizing participants’ own accounts. Although the literature indicated that symptom stabilization, therapeutic relationships, multidisciplinary collaboration, and post-discharge support may surround offense-related work, these domains were neither presented to participants nor used as a checklist, predetermined codes, or categories. The RNR model, GLM, and recovery-oriented perspectives likewise provided conceptual context without structuring the interview questions or coding. The two authors reviewed the guide for relevance and clarity. However, no independent review by qualitative researcher or forensic mental health nursing expert or pilot interview was conducted.

The semi-structured interviews explored the practices that participants considered part of support for self-reflection. All participants were asked the same broad opening question: “Apart from group self-reflection programs and individual interviews specifically related to self-reflection, what other forms of support do you consider to constitute support for self-reflection in forensic psychiatric wards?” Flexible, non-standardized follow-up questions were used to clarify the specific practice, context and timing, rationale for linking it to self-reflection, and distinction from general care. The research question, fixed opening question, and functions of the follow-up prompts are provided in Supplementary Material S1. Demographic data included age, self-identified gender, years of psychiatric nursing experience, and years of forensic psychiatric experience.

Data were collected between May and November 2025. The principal investigator or co-researcher conducted a single face-to-face interview with each participant in a private room at the participant’s hospital. Neither interviewer had a prior relationship with the participants. With written consent, interviews were audio-recorded digitally and transcribed verbatim in Japanese. Interviews lasted 40–80 min. No field notes were taken, and transcripts were not returned to participants for member checking.

No formal saturation threshold was specified in advance. Sample adequacy was assessed in relation to the focused descriptive aim and the breadth of accounts across six hospitals and varying levels of forensic nursing experience. After 10 interviews, the 11th did not yield substantively new nursing practices relevant to the research question. Two additional nurses, who had previously consented, were interviewed as planned, resulting in a final sample of 13. Recruitment ended after these scheduled interviews were completed, and all 13 accounts were included in the comparative analysis. Because variation was not deliberately maximized, the findings should be interpreted with caution.

### 2.4. Data Analysis and Rigor

Data were analyzed in Japanese using the approach to inductive qualitative content analysis described by Graneheim and Lundman [24]. This approach aligned with the qualitative descriptive aim. Coding focused primarily on manifest content, with limited interpretive abstraction to preserve context and develop clinically meaningful categories. The RNR model, GLM, and recovery-oriented perspectives were not used as coding frameworks or a priori categories; instead, they were applied only after the empirical categories had been finalized during the interpretation phase (Section 4).

The principal investigator conducted the initial analysis using a Microsoft Excel coding matrix. He repeatedly read each transcript to gain an overall understanding and extracted meaning units describing practices that participants linked to support for self-reflection outside formal programs and dedicated interviews. Routine activities, such as including symptom stabilization, daily-life support, discharge planning, and community coordination, were retained only when the participant’s account as a whole, read within its transcript context, explicitly connected them to preparing for, deepening, applying, or sustaining offense-related self-reflection. Descriptions of general care without this connection were excluded. Meaning units were condensed without losing contextual meaning and were coded using language close to the participants’ accounts.

The principal investigator compared codes for semantic similarity and relevance to the research question and grouped them into provisional subcategories. The co-researcher then reviewed the transcripts, meaning units, codes, and developing analytic structure. The researchers compared subcategories within and across transcripts, discussed alternative interpretations, and iteratively developed higher-order categories. Category labels and boundaries were repeatedly checked against the source text. Participants were not assigned to a single category; a single participant could contribute meaning units to multiple categories.

Category boundaries were defined by the function that a practice served in relation to self-reflection: (1) direct examination of the offense, its consequences, and responsibility; (2) establishment of clinical or relational conditions that enabled reflection; (3) translation of reflective insights into immediate prevention and self-management; (4) extension of reflective learning into post-discharge life planning; and (5) integration of offense-related understanding into shared inpatient–community planning. The final five categories were accepted when they were inherently coherent, functionally distinct, and able to accommodate all relevant coded material without forcing discrepant content into an unsuitable category.

Researcher reflexivity was considered throughout data collection and analysis. The principal investigator’s experience in community forensic mental health nursing sensitized him to continuity of care and community transition; nevertheless, it could also have predisposed him to conceptualize support too broadly. Conversely, the co-researcher’s lack of forensic nursing experience provided distance from field-specific assumptions, although it sometimes required additional contextual explanation. The broad opening question and participant-led follow-up prompts reduced the likelihood of directing participants toward the researchers’ expectations. During analysis, the researchers made their positions explicit, considered both narrower and broader interpretations, and returned to the source text whenever an interpretation appeared to reflect professional assumptions rather than the participants’ accounts.

To strengthen credibility and dependability, the co-researcher reviewed the complete analytic chain from transcripts and meaning units to codes, subcategories, and categories. Differences in interpretation prompted re-examination of transcript context and category boundaries rather than assessment of statistical inter-coder reliability [24,25]. Although no formal negative-case analysis was undertaken, contrasting accounts were retained when they clarified the conditions or limits of a category. A nursing professor with expertise in qualitative research conducted peer debriefings, and the researchers maintained an audit trail of coding and category revisions. Member checking was not conducted because the analytic aim was to identify patterns across participants’ accounts rather than to seek endorsement of individual transcripts or the final interpretation. Overall, co-researcher review provided complementary professional check of the analytic process, peer debriefing offered a methodologically informed critique of the analytic logic, and systematic transcript comparisons and the audit trail provided additional support.

All coding and category development were performed in Japanese. After finalizing the Japanese category structure and illustrative quotations, a professional translation service translated manuscript and category and subcategory labels, and selected quotations into English. The authors then compared the English wording with the Japanese originals and revised it when necessary to preserve conceptual and contextual meaning. Thus, translation occurred after analysis, not during it. Illustrative quotations were selected after the authors reread the full transcript context. Ellipses indicate omitted material, and square brackets provide only minimal referential or contextual clarification based on the immediately surrounding Japanese transcript, without introducing information or interpretations not found in the participant’s account.

### 2.5. Ethical Considerations

The Ethics Review Committee of Nara Medical University approved the study protocol on 1 November 2024 (protocol code 3884). Participants received verbal and written explanations of the study purpose and methods, the voluntary nature of participation, the right to withdraw, potential benefits and burdens, protection of personal information, and dissemination of findings. All participants provided written informed consent before data collection. Participant identifiers were replaced with study codes, identifying details were removed from transcripts and quotations, and research data were stored on a password-protected computer kept in a locked cabinet accessible only to the research team.

## 3. Results

Thirteen nurses from six forensic psychiatric hospitals participated. Eleven identified as men and two as women, and their ages ranged from 30 to 59 years. The mean psychiatric nursing experience was 14.5 (range, 2–35) years, and the mean experience in forensic psychiatric wards was 8.5 (range, 1–19) years. Participant characteristics are presented in Table 1. The analysis generated 5 categories and 15 subcategories describing functions participants attributed to nursing practices surrounding formal programs and dedicated interviews (Table 2). These categories are not presented as validated components of offense-related self-reflection.

### 3.1. Categories of Nurses’ Perceptions

The five categories were distinguished by their function in relation to reflection: direct offense-related content, enabling conditions, application to prevention, extension into future-life planning, and continuity through shared care planning. The categories were related but were neither mutually exclusive nor sequential, and one participant could contribute accounts to several categories. Broader nursing or rehabilitation activities were retained only when the participants explicitly linked them to the reflective process; otherwise, they were excluded. Some participants emphasized direct offense-focused dialogue, whereas others described a broader range of related practices. Contrasting accounts were retained when they clarified category boundaries. Associations among life history, symptoms, substance use, and offending behavior reflect participants’ clinical interpretations and do not establish causal relationships. For example, shopping or money management assistance was treated as general daily-life care when it was described only as helping the patient maintain everyday living and included only when the participant linked the activity to an offense-related vulnerability, warning sign, or prevention strategy identified through reflection.

#### 3.1.1. Examining Offenses, Victim Impact, and Responsibility

This category captured the direct, offense-focused content that participants regarded as support for self-reflection. It comprised five subcategories. Direct offense-focused content was included only when it arose in nursing practice surrounding formal interventions, such as ward-based follow-up after a program, review of program homework or records during routine care, spontaneous conversations in daily interactions, or visits to relevant locations undertaken as part of broader assessment or discharge preparation. Scheduled group programs and planned one-to-one offense-focused interviews were excluded. When offense-related material arose in a formal setting, only subsequent nursing follow-up or application outside the session was retained.

Reviewing the facts of the offense: After reviewing appraisal reports and clinical records, nurses worked with patients to reconstruct the course of the offense. They accompanied some patients to relevant locations, explored their mental state and circumstances at the time, and clarified details such as the location and extent of injuries. They also reviewed symptoms, medication use, and outpatient attendance around the time of the offense.

“I explained to the patient how many centimeters long the knife was, whom the patient stabbed, where the patient stabbed the person, and such details. The patient said that they had caused a terrible, selfish incident.”

(N8)

Linking offense background to the patient’s life history: Rather than treating the offense as an isolated event, nurses connected it to the patient’s life history, including upbringing, family relationships, attachment experiences, bullying, delinquency, and patterns of dependence. They explored possible associations between developmental or relational experiences and the patient’s later perceptions and behavior as clinical hypotheses, without reducing the offense to a single cause and presenting these associations as established causal explanations.

“The patient showed unusually intense reactions toward women. For example, the patient felt mocked simply by being looked at by a woman. After listening to the patient’s story, I thought that difficulties in the relationship with the mother might be connected to the later violence toward women, and I shared that perspective with the patient.”

(N7)

Considering the impact on victims and families: Nurses encouraged patients to consider the feelings of victims and recognize that the consequences of the offense extended to victims’ families, patients’ families, and others around them.

“I wanted the patient to understand the burden they had placed on their family. Because I had heard from the family about the difficulties they had experienced, I shared those experiences with the patient so that they could recognize the impact of their actions.”

(N2)

Clarifying apology, accountability, and responsibility: Nurses explored the concrete meaning of statements such as “I think I did something wrong” and “I am sorry.” They supported patients to consider accountability, possible reparation, and responsibility for preventing reoffending.

“I support patients’ self-reflection from the perspective of responsibility. For example, the words they offer to victims’ families are connected to their responsibility to apologize, provide compensation, and prevent reoffending.”

(N6)

Exploring justification and blame narratives without immediate rejection: Rather than immediately rejecting statements such as “the other person was at fault” or “I was not at fault,” nurses treated these accounts as material for further exploration. They considered whether delusions, persecutory beliefs, limited cognitive or emotional capacity, reluctance to disclose, or unresolved feelings shaped the patient’s narrative.

“The patient honestly told me, ‘I still have unresolved feelings toward the person against whom I committed the offense.’ At that time, I asked why the patient felt that way and encouraged the patient to express thoughts and feelings.”

(N4)

#### 3.1.2. Clinical and Relational Readiness for Self-Reflection

This category addressed the conditions that participants believed enabled patients to engage meaningfully in offense-related self-reflection. It comprised three subcategories. Clinical stabilization, daily-life structure, education, and relationship building were included only when participants explicitly connected them to the patient’s readiness or capacity for reflection.

Stabilizing mental state and daily life: Nurses viewed adequate judgment, mental-state stability, and an orderly daily life as conditions that enabled patients to examine the offense without becoming overwhelmed.

“For self-reflection to be possible, I think patients need to have good judgment, a stable mental state, and an orderly daily life. These conditions enable them to look back on and reflect on the offense. Therefore, we interact with patients in ways that provide a sense of safety and security so that they can engage in self-reflection without feeling overwhelmed.”

(N3)

Linking illness and substance-use education with self-reflection: Rather than presenting illness education as general knowledge, nurses connected it directly to each patient’s experiences. They supported patients in considering possible relationships among symptoms, treatment interruption, substance use, and behavior at the time of the offense. These relationships reflected participants’ clinical interpretations rather than causal findings of this study. Furthermore, some nurses viewed participation in addiction programs for subsequent offense-focused reflection.

“The offense was a reaction caused by symptoms. An illness education program is implemented before the self-reflection program, and we support patients so that they can realize that those reactions occurred because of symptoms.”

(N11)

Building a trusting therapeutic relationship: Nurses emphasized developing trust through ordinary interactions, such as informal conversation, shared activities, outings, and recreation. They sought to create relationships in which patients experienced the nurse as supportive and attentive, allowing difficult feedback to be offered without rupturing the therapeutic relationship.

“The premise is that we need to build a relationship in which the patient recognizes that this person is on their side … I am conscious of building trust so that even when I say something that gets to the heart of the matter, the patient does not come to dislike me.”

(N12)

#### 3.1.3. Applying Reflective Insights to Recidivism-Prevention Actions

This category described how participants translated reflective understanding into concrete prevention and self-management actions. It comprised two subcategories.

Incorporating insights into crisis and self-monitoring plans: Nurses incorporated warning signs and coping strategies identified through self-reflection into crisis plans and self-monitoring tools. When a patient’s reflective understanding remained limited, nurses emphasized system-level safeguards, such as early unscheduled outpatient contact or short-term hospitalization. They also helped patients monitor sleep, physical symptoms, as-needed medication use, and other changes to recognize concerning patterns and seek sought early.

“It is also important to incorporate the knowledge gained through the self-reflection program and the methods learned for preventing reoffending into the crisis plan.”

(N7)

Applying insights to daily-life risk management: Daily-life risk management, including money management, meals, medication, and daily rhythm, was included only when nurses explicitly connected these routines to vulnerabilities or circumstances preceding the offense and to practical actions intended to prevent recurrence.

“Patients learn that disruptions in their daily lives can lead to a worsening of their condition. As a result, they come to understand that they may reoffend. … When patients are discharged, they need to ensure that they do not reoffend. To support this, we provide education to help them understand the importance of maintaining a structured daily life, including money management, healthy eating, medication adherence, and adequate sleep.”

(N2)

#### 3.1.4. Extending Reflective Learning into Post-Discharge Life Planning

This category described how participants extended offense-related learning into plans for where, with whom, and how the patient would live after discharge. It comprised three subcategories. General housing, service, or recovery planning was insufficient for inclusion; participants had to explicitly connect these plans to vulnerabilities, coping strategies, or meanings identified through self-reflection.

Linking offense-related vulnerabilities to living environments: Housing and environmental conditions were included when participants connected them to offense-related vulnerabilities or relapse and risk patterns identified through reflection. Examples included avoiding environments associated with substance use and assessing noise or overstimulation that might exacerbate symptoms relevant to risk.

“While reviewing the offense with the patient, we learned that there was someone near the patient’s former home who could sell them drugs. The patient had acquaintances who were involved in drug trafficking, so I recommended that the patient relocate to a different area after discharge.”

(N12)

“When visiting the proposed post-discharge setting, we ask questions such as, ‘What about that kind of noise?’ and ‘Does it bother you?’ I consider such discussions to be part of support for self-reflection.”

(N10)

Translating reflective learning into post-discharge routines and supports: Nurses worked with patients to make coping and prevention strategies identified through reflection feasible within the intended living environment. Money management, shopping, meals, medication, and vocational or social services were included only when they operationalized these strategies; general assistance with daily life was excluded.

“During the self-reflection program, the patient described how difficulties in managing money had led to a breakdown in daily living and a worsening of their condition, which they perceived as contributing to the offense … The patient’s money management deteriorated to the point that the patient could no longer shop for food or manage everyday living. Therefore, we supported the patient in accessing community support services that could provide comprehensive assistance with daily living, including money management.”

(N2)

“Personally, I think that coping methods to prevent reoffending are an important of support for self-reflection. They cannot be made concrete during hospitalization, but we discuss how it would be best to cope after discharge to prevent reoffending.”

(N10)

Connecting self-reflection with hope, strengths, meaning, and future goals: Future-oriented planning was included as support for self-reflection only when patients linked reflection on the offense and experiences with their hopes, interests, strengths, work aspirations, social participation, and preferred ways of living. General recovery planning without this explicit connection was excluded.

“To live well, I think it is important to look back on the past, but we also need to help patients move forward with hope and connect that reflection to how they will live their lives in the future.”

(N1)

“Rather than ending with reflection on the offense alone, we discuss with patients how they can draw on their own strengths to move forward and live their lives.”

(N1)

#### 3.1.5. Integrating Offense-Related Understanding into Inpatient–Community Care Planning

This category described how offense-related understanding and its practical implications were carried into plans shared among patients, multidisciplinary professionals, and community providers. It comprised two subcategories. General coordination or patient participation was excluded unless explicitly linked to offense-related reflection, warning signs, coping strategies, or related future goals.

Transferring offense-related understanding to community providers: Nurses communicated patients’ understandings of the offense, views of responsibility, warning signs, coping strategies, and related life concerns to community services, such as home-visit nursing, day care, vocational support, and outpatient care. This information was translated into post-discharge community support.

“In the self-reflection program, patients also learn about the need to prevent reoffending. We develop a shared understanding with patients that receiving support from community providers can help prevent reoffending. … I consider support for self-reflection to include sharing with the people who will support the patient after discharge things such as the use of home-visit nursing after discharge, ways to relieve stress, and what the patient’s warning signs are.”

(N8)

Coordinating multidisciplinary planning and patient participation: Multidisciplinary planning and patient participation were included only when linked to applying self-reflection to illness management and future-life planning. Nurses shared daily-life observations, coordinated with psychologists and other professionals, incorporated patients’ wishes into meetings and support plans, and supported them in expressing their future goals. General preference elicitation or participation without this reflective connection was excluded.

“[Through support for self-reflection,] patients come to understand that unless they take an active role in planning their own lives, they may not be able to manage their illness … Patients gradually come to understand that managing their own illness can help prevent reoffending … It is common for supporters to take the lead in developing support plans at meetings, but we listen to the patient’s wishes and incorporate them into the support plan. Also, at the meeting, we ask the patient to describe to the care team the kind of life they want to lead.”

(N13)

## 4. Discussion

In this study, we examined how nurses in Japanese MTSA inpatient wards perceived the scope of support for offense-related self-reflection outside formal group programs and dedicated interviews. The five categories represent functions that participants attributed to practices beyond these formal interventions: direct examination of the offense, preparation for reflection, application of reflective learning to prevention, extension into post-discharge planning, and continuity through shared care planning. The categories should not be interpreted as inherent or effective components of self-reflection. Consistent with the qualitative descriptive design, they represent a context-bound account of nurses’ perceptions rather than a formal theory, objective taxonomy, or universal sequence of care.

### 4.1. Conceptual Boundaries Beyond Formal Programs and Dedicated Interviews

Offense-related treatment may include examining the offense and its background and linking this understanding to treatment and psychosocial support [4]. Previous studies have described offense-focused work within the nurse–patient relationship [10], therapeutic relationship work in secure settings [11,12], patient engagement [13], shared risk assessment [14], and clinical reasoning in forensic nursing [15]. The present findings do not classify all such activities as self-reflection; rather, they described the circumstances under which they perceived surrounding nursing practices as relevant: when the practice prepared patients to reflect, deepened offense-related understanding, translated learning into action, or carried that understanding into future care.

The categories were functionally distinct but interconnected. Clinical stabilization and trust served as enabling conditions, whereas risk-management actions represented applications of reflective learning. Post-discharge and community activities entered the findings only when participants explicitly connected them to offense-related vulnerabilities, coping strategies, or meanings identified through reflection. Thus, the findings describe perceived connections around formal interventions and do not imply that routine nursing or rehabilitation activities inherently constitute support for self-reflection.

Although formal offense-specific group programs remain important, their evidence base and integration into routine practice are still developing [9]. The five categories may help clarify nursing practices surrounding formal interventions and inform future research. However, their clinical relevance and effects require further evaluation.

The breadth of the findings may also reflect the Japanese MTSA context. Designated inpatient services combine structured treatment, continuous ward-based nursing, multidisciplinary planning, and transition to coordinated community services [19,20,21]. Japanese community nursing research has similarly identified links among offense-related work, risk management, transition, legal understanding, and multidisciplinary coordination [20]. This integrated pathway may have contributed to the five-category structure. Its transferability to systems with different nursing roles, program structures, or arrangements for community follow-up requires further study.

### 4.2. Examining Offenses, Victim Impact, and Responsibility

Addressing an offense has been described as examining the events, situations, behaviors, and choices associated with it and locating them within the person’s life narrative [4]. Participants likewise situated the offense within both concrete events and broader life histories. Their accounts included clinical hypotheses about possible associations among symptoms, treatment interruption, family and relational experiences, substance use, and interpersonal patterns. These findings do not establish causal relationships. Nevertheless, this direct offense-focused category is consistent with previous descriptions of therapeutically addressing criminal offenses within the nurse–patient relationship [10].

Participants’ attention to the impact on victims and families and to the meaning of apology, accountability, and responsibility reflected efforts to address both treatment and public-safety responsibilities [1,26]. Moreover, their willingness to explore justification and blame narratives without immediate rejection reflected an effort to avoid punitive interactions. These findings suggest distinguishing accountability-oriented dialogue from coercive demands for remorse and pacing according to the patient’s cognitive, emotional, and psychiatric capacities.

### 4.3. Clinical and Relational Readiness for Self-Reflection

Participants viewed mental-state stability, daily-life organization, illness understanding, and treatment engagement as conditions that could help patients tolerate offense-related reflection. Reviewing an offense may evoke anxiety, guilt, shame, and defensive responses [27,28]. Participants also linked symptoms, treatment interruption, disengagement from outpatient care, or substance use to the offense as part of their clinical formulations. Although such formulations may serve explanatory or preventive purposes in care, they do not demonstrate causation.

Participants also perceived a trusting therapeutic relationship as necessary for reflection. Staff, environmental, and organizational factors may influence therapeutic relationships in secure services [11], which may require deliberate rebuilding after coercive or restrictive events [12]. Relational security emphasizes safety developed through knowledge of and engagement with the patient [29]. Participants’ emphasis on informal conversation, shared activities, and reliable contact indicates that the capacity to discuss difficult offense-related material may develop through ordinary nursing encounters.

Symptoms, paternalistic practices, and power imbalances may limit patient engagement in forensic mental health care [13]. In participants’ accounts, readiness for self-reflection therefore required both clinical stability and a relationship in which the patient could speak safely. In this study, we did not evaluate whether these relational practices improved engagement or reflective capacity.

### 4.4. Applying Reflective Learning to Risk Management and Post-Discharge Planning

The RNR model emphasizes intervention on dynamic needs associated with recidivism risk [2,8]. Participants described incorporating reflective insights into crisis plans, self-monitoring, help-seeking, medication and sleep routines, substance-use management, money management, and daily rhythm. This interpretation is consistent with collaborative approaches to risk assessment and management [14,30]. However, the present study demonstrates only that nurses perceived these practices as applications of reflective learning; we did not test whether they reduced risk or recidivism.

Forensic psychiatric nursing requires clinical reasoning that considers the offense, risk, legal context, and therapeutic relationship [15]. Because nurses observe patients across everyday situations, participants believed that they could compare what patients expressed during reflective dialogue with their behavior in daily life and help translate abstract intentions into feasible actions. This represents a perceived function of practice rather than evidence of effectiveness.

Post-discharge planning was included in the findings only when participants anchored it in reflective learning. They linked living environments, routines, services, strengths, and future goals to vulnerabilities, warning signs, coping strategies, or personal meanings identified through reflection. The GLM and recovery literature provide an interpretive context for valued living, hope, identity, participation, and safety [5,6,7,8,16,17,18,31,32,33]. These perspectives do not imply that housing, vocational support, or recovery planning are inherently components of offense-related self-reflection. Rather, participants perceived these activities as relevant when they extended reflection into a feasible and sustainable plan for future life.

### 4.5. Inpatient–Community Continuity in the Japanese MTSA Context

Participants described communicating offense-related understandings, warning signs, coping strategies, and related life concerns to community providers. Continuity is particularly relevant in the MTSA pathway, in which inpatient treatment is linked to coordinated outpatient and community support [19,20,21]. The practical value of reflection developed during hospitalization may depend, in part, on whether these understandings can be carried forward after discharge; however, we did not directly evaluate continuity or outcomes in the present study.

Participants also described integrating reflective learning and patients’ future goals into multidisciplinary planning. Patient participation was explicitly situated within support for self-reflection and linked to taking an active role in illness management and life planning. This category therefore concerns incorporating reflective understanding into shared care planning rather than simply eliciting preferences. Patient engagement requires action across patient, staff, process, organizational, and sociopolitical levels [13]. Within participants’ accounts, translating reflective understanding into collaborative planning represented a potential nursing contribution to continuity. Because the study did not compare professions, it cannot establish whether this contribution is unique to nursing.

### 4.6. Implications for Forensic Mental Health Nursing Practice and Research

For clinical practice, the five categories can be used as structured prompts for nursing assessment, case formulation, and care planning rather than as a checklist of mandatory interventions. In individual contacts and case conferences, nurses can ask whether (1) the patient is able and willing to examine the offense and its impact; (2) mental-state stability, daily-life structure, and a sufficiently trusting relationship are in place; (3) insights from reflection have been translated into individualized warning signs, coping responses, help-seeking steps, and daily routines; (4) these strategies have been adapted to the intended living environment and linked to the patient’s strengths and future goals; and (5) the resulting understanding has been incorporated into a shared inpatient–community plan. Documenting the connection between each nursing action and the reflective process may help distinguish focused support from routine psychiatric nursing, rehabilitation, or discharge planning.

Before initiating or deepening offense-related dialogue, nurses should assess the patient’s readiness and adjust the timing and pace to the patient’s mental state, cognitive and emotional capacity, and level of trust. Routine ward interactions can then help determine whether reflective insights are evident in daily behavior and support rehearsal of concrete actions, such as monitoring warning signs, maintaining sleep and medication routines, seeking help early, managing money or substance-related risks, and avoiding high-risk environments. Accountability-oriented conversations should explore the meaning of apology and responsibility without demanding remorse or immediately rejecting defensive or blame-based narratives. Clinical supervision is crucial to help nurses balance therapeutic engagement, patient autonomy, and public-safety responsibilities.

Before discharge, nurses can work with the patient and multidisciplinary team to prepare a concise summary, co-developed with and understood by the patient, of offense-related vulnerabilities, early warning signs, coping strategies, preferred supports, environmental risks, strengths, and future goals. Subject to relevant legal and ethical requirements for information sharing, this summary can facilitate handovers to outpatient, home-visit nursing, vocational, and other community services. Education for forensic mental health nurses should therefore include non-punitive offense-related dialogue, readiness assessment, translation of reflection into crisis and daily-life plans, and collaborative inpatient–community planning.

The five categories also provide an initial framework for research; however, they are not a standardized intervention, a validated construct, or evidence of effectiveness. Thus, further studies should involve patients, families, multidisciplinary professionals, and community providers; observe how the proposed functions are enacted in practice; examine the clarity and acceptability of the suggested practice prompts; refine category boundaries; and evaluate processes and outcomes important to patients.

### 4.7. Limitations

The transferability of the findings is limited by the qualitative design, specific Japanese MTSA context, and convenience sampling strategy. Although participants varied in age and experience, this variation occurred incidentally rather than through purposive maximum-variation sampling. Of the 13 participants, 11 identified as male, and the group was relatively experienced in psychiatric and forensic nursing. Recruitment through directors of nursing may also have favored nurses who were viewed positively by management or were particularly interested in the topic. Thus, the categories may reflect the practices and organizational arrangements of the participating hospitals and may not represent all Japanese forensic psychiatric settings or other legal and service systems.

The findings describe nurses’ reported perceptions rather than directly observed practice or demonstrated outcomes. Participants’ accounts may have been influenced by recall, social desirability, and professional terminology. The interview guide was reviewed only by the two authors; it was not independently reviewed by a qualitative researcher or forensic mental health nursing expert and was not pilot-tested. In addition, patients, family members, community providers, or other professional groups were not included, and effects on reflection, engagement, recovery, risk management, recidivism, or community living were not evaluated. Thus, the findings cannot establish the consistency, patient experience, or effectiveness of the described practices.

The broad opening question may have introduced conceptual ambiguity. Nurse participants may have found it challenging to distinguish support intended to facilitate offense-related self-reflection from routine nursing, rehabilitation, discharge planning, or multidisciplinary risk-management activities. Although the inclusion criteria required an explicit connection to the reflective process, some overlap may remain. Associations among life experiences, symptoms, substance use, and offending behavior represent participants’ clinical interpretations rather than causal findings, and translation from Japanese to English may have reduced some linguistic or cultural nuance. Future studies should refine the working definition; use independently reviewed, pilot-tested interview guides with concrete examples of inclusion and exclusion; seek divergent or negative cases; include patient and multidisciplinary perspectives; and test the framework through direct observation and in settings beyond the Japanese MTSA system.

## 5. Conclusions

Participants described five functions through which they perceived nursing practices surrounding formal programs and dedicated interviews to support offense-related self-reflection: direct examination of the offense; clinical and relational preparation; application of reflective learning to prevention; extension into post-discharge life planning; and integration into shared inpatient–community care planning. When used with caution, this framework may help nurses connect reflective dialogue with everyday ward care, individualized prevention planning, discharge preparation, and community handover. However, the categories summarize nurses’ perceptions in Japanese MTSA inpatient settings and do not establish that routine-care activities constitute self-reflection support or the practices are effective. Further validation should incorporate the perspectives of patients and other providers and examine in additional settings.

## Figures and Tables

**Table 1:** Participant characteristics.

Participant ID	Gender	Age, Years	Psychiatric Nursing Experience, Years	Forensic Psychiatric Ward Experience, Years
N1	Man	50–59	15	10
N2	Man	30–39	16	6
N3	Woman	50–59	18	13
N4	Man	30–39	9	3
N5	Man	30–39	11	2
N6	Man	30–39	10	4
N7	Woman	40–49	18	10
N8	Man	40–49	15	7
N9	Man	50–59	35	19
N10	Man	40–49	19	19
N11	Man	30–39	5	1
N12	Man	30–39	15	15
N13	Man	30–39	2	2

**Table 2:** Categories and subcategories of nursing support for offense-related self-reflection beyond formal programs and dedicated interviews.

Category	Subcategory
Examining offenses, victim impact, and responsibility	Reviewing the facts of the offense Linking offense background to the patient’s life history Considering the impact on victims and families Clarifying apology, accountability, and responsibility Exploring justification and blame narratives without immediate rejection
Clinical and relational readiness for self-reflection	Stabilizing mental state and daily life Linking illness and substance-use education with self-reflection Building a trusting therapeutic relationship
Applying reflective insights to recidivism-prevention actions	Incorporating insights into crisis and self-monitoring plans Applying insights to daily-life risk management
Extending reflective learning into post-discharge life planning	Linking offense-related vulnerabilities to living environments Translating reflective learning into post-discharge routines and supports Connecting self-reflection with hope, strengths, meaning, and future goals
Integrating offense-related understanding into inpatient–community care planning	Transferring offense-related understanding to community providers Coordinating multidisciplinary planning and patient participation

Note: The categories represent functions attributed by participants. Broader nursing, rehabilitation, or community-care activities were included only when participants’ full accounts explicitly linked them to preparing for, deepening, applying, or sustaining offense-related self-reflection.

## Data Availability

The qualitative interview data are not publicly available and cannot be shared upon request because participants did not consent to data sharing and the approved consent procedures and ethics protocol do not permit disclosure of the sensitive, potentially identifiable interview data.

## Supplementary Materials

The following supporting information can be downloaded at: https://www.mdpi.com/article/10.3390/nursrep16090313/s1, Supplementary Material S1: Semi-Structured Interview Guide.

## Public Involvement Statement

No public involvement in any aspect of this research.

## Guidelines and Standards Statement

This study is reported in accordance with the Standards for Reporting Qualitative Research (SRQR) [23].
