# Supplementary material for: Nurses’ Perceptions of the Scope of Nursing Support for Offense-Related Self-Reflection Beyond Formal Programs and Dedicated Interviews in Japanese Forensic Psychiatric Care
Source: Nurs Rep. 2026 Sep 2;16(9):313. doi: 10.3390/nursrep16090313 (PMC13610853; doi:10.3390/nursrep16090313)
Supplement: Supplementary file 1 [file nursrep-16-00313-s001.zip › nursrep-4496351-supplementary.pdf]

Semi-Structured Interview Guide

Nurses' Perceptions of the Scope of Nursing Support for Offense-Related Self-Reflection Beyond Formal Programs and Dedicated Interviews in Japanese Forensic Psychiatric Care

Purpose and Administration

Central Research Question

What nursing practices do nurses in Japanese MTSA inpatient wards perceive as supporting offense-related self-reflection beyond formal group programs and dedicated offense-focused interviews?

The guide was designed to address the study's central research question and elicit participants' own perceptions of the range of nursing support for offense-related self-reflection beyond formal group programs and dedicated offense-focused interviews. One broad opening question was asked of all participants. Follow-up prompts were non-standardized: their wording, order, and use varied according to each participant's response. Literature-derived domains were not read to participants as a checklist and were not used as predetermined analytical categories.

Guide Review and Pilot Testing

The two authors reviewed the guide for relevance and clarity. It was not reviewed by an independent qualitative researcher or forensic mental health nursing expert, and no pilot interview was conducted before data collection.

Fixed Opening Question

Original Japanese:

司法精神科病棟で行われている内省支援に関連して、集団内省プログラムや内省支援に関する個別面接以外に、どのような支援を内省支援に含まれるものと考えますか。

English translation:

Apart from group self-reflection programs and individual interviews specifically related to self-reflection, what other forms of support do you consider to constitute support for self-reflection in forensic psychiatric wards?

Functions of Non-Standardized Follow-Up Prompts

Depending on the participant's response, the interviewer used follow-up prompts to elicit clarification or elaboration concerning the following functions. These were not fixed verbatim questions, and not every function was used in every interview.

•Clarify the concrete nursing practice or interaction being described.

- Clarify the clinical situation, timing, and persons involved.
- Explore why the participant considered the practice to constitute support for self-reflection.
- Explore how the practice was perceived to enable, deepen, apply, or sustain offense-related self-reflection.
- Request a concrete example of the patient’s response or of how the practice was implemented.
- Clarify connections with everyday ward life, risk prevention, discharge planning, or continuity of community support when these were raised by the participant.
- Clarify perceived boundaries, including activities the participant would not consider part of support for self-reflection.

Note. Interviews and analysis were conducted in Japanese. The English version was prepared after the Japanese interview guide and analytical categories had been finalized, and the authors checked the wording against the Japanese original for conceptual equivalence.
